# Vision Improvement after Osimertinib Treatment in Paraneoplastic Optic Neuropathy Associated with Lung Adenocarcinoma

**DOI:** 10.1155/2021/2832021

**Published:** 2021-07-15

**Authors:** Masaomi Kubota, Nobumasa Tamura, Takaaki Hayashi, Euido Nishijima, Haruhiko Yanagisawa, Akira Kojima, Tadashi Nakano

**Affiliations:** ^1^Department of Ophthalmology, Katsushika Medical Center, The Jikei University School of Medicine, 6-41-2, Aoto, Katsushika-ku, Tokyo 125-8506, Japan; ^2^Department of Respiratory Medicine, Katsushika Medical Center, The Jikei University School of Medicine, 6-41-2, Aoto, Katsushika-ku, Tokyo 125-8506, Japan; ^3^Department of Ophthalmology, The Jikei University School of Medicine, 3-25-8, Nishi-shimbashi, Minato-ku, Tokyo 105-8461, Japan

## Abstract

Treatments for paraneoplastic optic neuropathy (PON), a tumor-related autoimmune disease, include immunosuppression, plasma exchange, and immunoglobulin therapies, as well as treatment of the underlying disease. Herein, we describe the clinical course of an older adult patient with PON whose loss of vision improved after switching between epidermal growth factor receptor-tyrosine kinase inhibitor (EGFR-TKI) treatments for cancer. A 76-year-old woman, who had been treated with gefitinib for lung adenocarcinoma for two years, presented with acute bilateral visual disturbances. Her decimal best-corrected visual acuity (BCVA) was 0.3 in the right eye (RE) and 0.7 in the left eye (LE). Slit-lamp examination and funduscopy showed no abnormal findings. Two weeks later, her BCVA decreased to 0.2 in the RE and 0.01 in the LE. Goldman's perimetry showed a defect in the lower nasal RE and extensive visual-field loss in the LE. Single-flash electroretinograms showed normal amplitudes. Magnetic resonance imaging revealed left optic neuritis and showed neither metastatic cancer nor multiple sclerosis. Pattern-reversal visual evoked potentials showed decreased P100 amplitudes in both eyes (BE). Based on a diagnosis of PON from clinical findings, methylprednisolone pulse treatment was administered. However, her BCVA became no light perception in BE two months after the first visit. Because the tumor tissue was found to be positive for the EGFR T790M resistance mutation by bronchoscopy, the EGFR-TKI treatment was changed to osimertinib, decreasing the size of the lung cancer lesions. Her BCVA improved to hand motion in BE. Her final BCVA was 0.01 in the RE, counting fingers 10 cm in the LE. She died at the age of 79 years. To our knowledge, no reports have shown improvement in BCVA in patients with PON after changing EGFR-TKI treatments. This report indicates that some patients may develop severe visual dysfunction without early treatment for the primary tumor.

## 1. Introduction

Paraneoplastic optic neuropathy (PON) is one of several tumor-related autoimmune diseases, such as cancer-associated retinopathy (CAR) and melanoma-associated retinopathy (MAR) [1, 2]. Malignant tumors, such as small-cell lung cancer and malignant lymphoma, are known to cause PON [1, 2], but it can also be caused by benign tumors, such as choroid meningioma [3]. Patients with PON usually exhibit bilateral subacute vision loss and cerebellar ataxia, and other neurologic deficits are seen in some patients [2]. It has been reported that anti-CV2/collapsin response-mediator protein-5 (CRMP-5) antibody is detected in PON associated with small-cell lung cancer [2]. In some patients with autoimmune optic neuropathies, visual function can be improved with corticosteroid treatment, and overall visual prognosis is better compared to those of CAR and MAR [1]. However, visual prognosis of PON depends upon responsiveness to treatment for the underlying malignancies [4, 5]. Herein, we describe the clinical course of an older adult patient diagnosed with and treated for PON associated with lung adenocarcinoma.

## 2. Case Presentation

A 76-year-old woman presented with acute bilateral visual disturbances starting one week prior to the first visit. She had been diagnosed with advanced stage 4 lung adenocarcinoma two years prior and treated with the first-generation epidermal growth factor receptor-tyrosine kinase inhibitor (EGFR-TKI), gefitinib, due to a somatic EGFR exon-19 deletion mutation.

**Figure 1:**
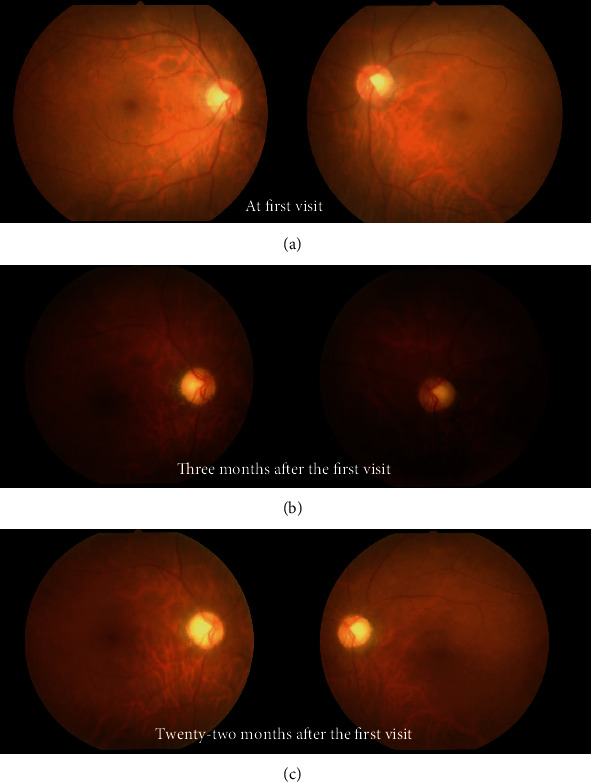
Fundus photographs. (a) At the first visit, funduscopy showed an almost normal appearance in both eyes. (b) Three months after the first visit, funduscopy showed optic disc pallor in both eyes. (c) Twenty-two months after the first visit, the optic disc pallor remained unchanged.

Her decimal best-corrected visual acuity (BCVA) was 0.3 in the right eye (RE) and 0.7 in the left eye (LE). Slit-lamp examination showed mild cataracts and no inflammatory cells in the anterior chamber and media. Funduscopy revealed no abnormal findings in the optic disc, retina, or vitreous humor in both eyes (BE) (Figure 1(a)). One week after presentation, her BCVA was decreased to 0.15 in the LE with a relative afferent pupillary defect.

**Figure 2:**
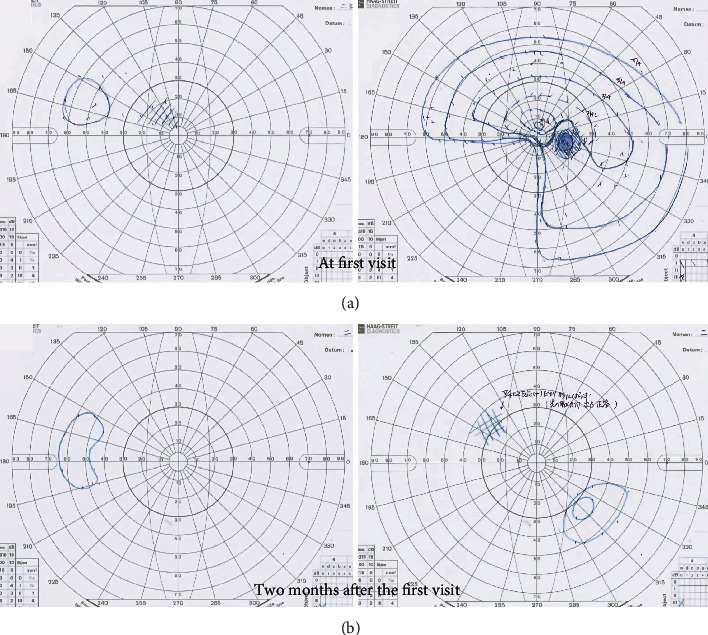
Visual fields. (a) At the first visit, Goldman's perimetry (GP) showed a visual-field defect of the lower nasal right eye and extensive visual-field loss in the left eye. (b) Two months after the first visit, GP showed severe visual-field defects with peripheral visual islands in both eyes.

**Figure 3:**
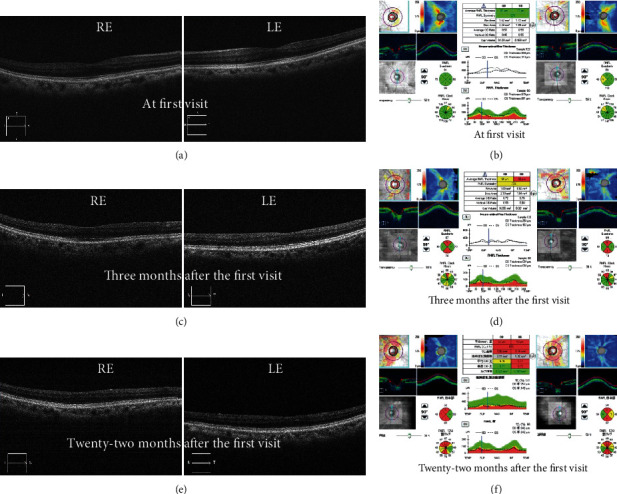
Optical coherence tomography (OCT) imaging. At the first visit, OCT revealed no remarkable findings on the macula and optic discs in both eyes (a, b). However, the macula (c, e) and optic disc head (d, f) had thinned in both eyes during the clinical course.

**Figure 4:**
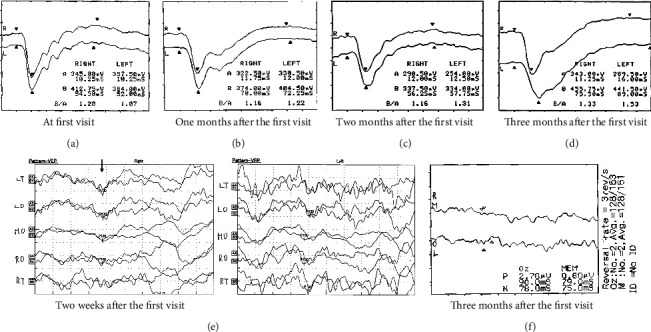
Dark-adapted single-flash electroretinograms (ERG) and pattern visual evoked potential (VEP) findings. (a) At the first visit, ERG showed normal amplitudes in both eyes (BE). (b) One month after the first visit, ERG showed slightly decreased oscillatory potentials (OPs) in BE. (c) After changing the EGFR-TKI treatment, the ERG response did not change in BE two months after the first visit. (d) Three months after the first visit, ERG showed markedly decreased OPs in BE. (e) VEP two weeks after the first visit had decreased P100 amplitudes in the midline occipital region of BE. (f) VEPs are non-recordable in BE.

**Figure 5:**
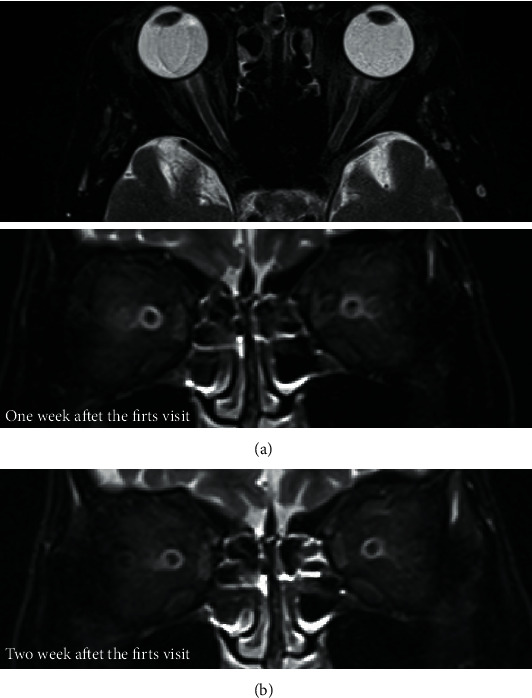
Magnetic resonance imaging (MRI). (a) One week after the first visit, T2-weighted MRI demonstrates well-marked enhancement of the left optic nerve compared to the right optic nerve. (b) Two months after the first visit, MRI showed improvement in the enhancement of the left optic nerve.

One week later, her BCVA was severely decreased to 0.2 in the RE and 0.01 in the LE. Goldman's perimetry (GP; Haag-Streit, Bern, Switzerland) showed a visual-field defect in the lower nasal RE and extensive visual-field loss in the LE (Figure 2(a)). However, spectral-domain optical coherence tomography (OCT; Carl Zeiss Meditec AG, Dublin, CA, USA) revealed no remarkable findings in all retinal layers nor swelling around the optic discs in BE (Figures 3(a) and 3(b)). Dark-adapted single-flash electroretinograms (ERG; LE-4000, Tomey, Nagoya, Japan) using a strong flash (200 cd s m^−2^) showed normal amplitudes in BE (Figure 4(a)). We consulted neurologists for the possibility of retrobulbar optic neuritis, multiple sclerosis, or neuromyelitis optica. Brain and orbital magnetic resonance imaging (MRI) revealed left optic neuritis but showed neither metastatic cancer nor the white matter lesions seen in multiple sclerosis (Figure 5(a)). Cerebrospinal fluid examination revealed no remarkable findings. Full-field pattern-reversal visual evoked potentials (VEPs) showed decreased P100 amplitudes in BE, indicating bilateral optic neuropathy (Figure 4(e)).

Three weeks after the first visit, her BCVA had further decreased to 0.02 in the RE and no light perception (NLP) in the LE, and ERG showed slightly decreased oscillatory potentials (OPs) in BE (Figure 4(b)). Treatment with oral prednisolone was started at 30 mg per day and gradually tapered, but there was no improvement in visual function. Her BCVA decreased to light perception (LP) in the RE and NLP in the LE after the first treatment, and to NLP bilaterally one week later with dilated pupil and absence of the pupillary light reflex. Subsequently, three courses of methylprednisolone (1,000 mg per day) pulse treatment was administered for 3 days but her visual acuity did not improve; her BCVA remained NLP bilaterally at two months after the first visit.

Serum autoantibody tests were negative for anti-aquaporin-4 and anti-myelin-oligodendrocyte glycoprotein (MOG) by enzyme-linked immunosorbent assay. Autoantibodies, such as anti-Tr/DNER, anti-GAD65, anti-ZIC4, anti-TITIN, anti-SX1, anti-recoverin, anti-Hu, anti-Yo, anti-Ri, anti-PNMA (Ma2/Ta), anti-CV2/CRMP-5, and amphiphysin antibodies, were negative by immunochromatography analysis. Clinically, PON was diagnosed by exclusion diagnosis.

As transbronchial lung biopsy of the primary lesion revealed the acquired EGFR T790M resistance mutation, her EGFR-TKI treatment was changed from gefitinib to osimertinib, a third-generation EGFR-TKI. Subsequently, her primary and metastatic lung cancer lesions decreased in size.

One week after the change in EGFR-TKI treatment, her BCVA had improved to hand motion in BE. Two months after the first visit, GP showed severe visual-field defects but recovered peripheral visual islands in BE (Figure 2(b)). Orbital MRI showed improvement in the enhanced left optic nerve (Figure 5(b)); however, the ERG response had not changed in BE (Figure 4(c)).

Three months after the first visit, her BCVA further improved to counting fingers (CF) 20 cm in BE. However, pattern-reversal VEPs were nonrecordable in BE. The ERG showed markedly decreased OPs in BE (Figure 4(f)). Funduscopy revealed optic disc pallor (Figure 1(b)), and OCT showed thinning of all layers of the macula and the neuroretinal rim of the optic nerve head in BE (Figures 3(c) and 3(d)), although there was improvement of visual acuity (from NLP to CF). Additional MRI scans (Figure 5(b)) performed during the course of the disease showed no evidence of optic neuropathy or optic nerve abnormalities.

**Figure 6:**
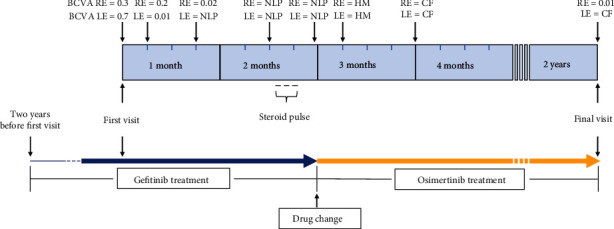
Visual acuity and treatment timeline. Decimal best-corrected visual acuity (BCVA) is described from the first visit to the final visit. RE: right eye; LE: left eye; CF: counting fingers; HM: hand motion; NLP: no light perception. Gefitinib is changed to osimertinib two months after the first visit. Three courses of steroid pulse treatment are administered before changing to osimertinib.

At her final visit, approximately two years after the first visit, her BCVA remained 0.01 in the RE and CF 10 cm in the LE, and funduscopy showed optic disc pallor in BE (Figure 1(c)). OCT revealed the thinned macula and optic nerve head (Figures 3(e) and 3(f)). Visual acuity and treatment timeline were described in Figure 6. She died at the age of 79 years.

## 3. Discussion

In this report, we describe a rare case of clinically diagnosed PON associated with lung adenocarcinoma in which visual acuity was initially improved after switching between EGFR-TKI treatments, but ultimately resulted in severe loss of visual acuity. To our knowledge, no reports have demonstrated improvement in visual acuity by changing EGFR-TKI treatments during the treatment of PON.

The differential diagnosis of a disease that causes rapid vision loss with no findings in the optic disc or retina at the first visit includes posterior ischemic optic neuropathy (PION), CAR, and PON. PION was excluded because our patient had none of the risk factors (e.g., coagulopathies; ischemic heart disease, such as angina or myocardial infarction; and stenosis of the internal carotid artery) and no inflammation; moreover, the onset was subacute and binocular, and the course was progressive.

CAR and MAR are well known for progressive retinal degeneration, which is a frequent complication in patients with cancer, and an absence of obvious abnormalities by funduscopy at the first consultation. Anti-recoverin antibodies are often detected in CAR and anti-bipolar cell antibodies (e.g., anti-TRPM1) in MAR as specific serum autoantibodies. Further, ERG shows extremely reduced a- and b-wave responses in CAR and a negative-ERG pattern in MAR. However, in our patient, CAR and MAR were excluded based on the normal ERG findings, lack of night blindness, and absence of anti-recoverin antibodies at the first visit. Furthermore, immune-mediated optic neuritis, such as MS and NMO, were ruled out as tests were negative for anti-aquaporin antibody and anti-MOG antibody. We established PON as exclusion diagnosis based on clinical findings in total. On the other hand, the T2 signal of MRI in LE at the first visit of this patient was increased. This may have been an inflammatory process, but there is also a case report of increased signal on MRI in cases of PON [6]. In this case, an increase in the signal of the left eye on the MRI T2 image was considered to be consistent with the diagnosis of PON. The enhancement of the RE is unknown because no contrast-enhanced MRI was performed, but the reason that the increase in signal was unilateral was that the onset was predominantly in the LE, with marked loss of visual acuity. It is possible that the signal increase in the RE was minimal on T2-weighted MRI.

PON is an autoimmune optic neuropathy, and CRMP-5 has been most frequently reported as an autoantigen associated with PON [2, 7–9]. Anti-CRMP-5-positive PON develops as optic neuritis with anterior vitreous cells, optic disc swelling, and leakage of the optic disc on fluorescein angiography [4, 5]. On the other hand, some patients with PON do not have swelling of the optic disc [8]. Although the detection of anti-CRMP-5 gives a definitive diagnosis of PON, most patients with PON and anti-CRMP-5 antibodies have small-cell lung cancer. There are few reports of specific autoantibodies in PON due to lung adenocarcinoma [10]. In this case, serum autoantibody testing, including anti-CRMP-5, was negative and no retinal/vitreous inflammation nor optic disc swelling was observed during the clinical course. However, PON was diagnosed based on the history of lung adenocarcinoma and on clinical examination.

Because PON is an autoimmune disease, corticosteroid therapy is the first-line treatment, and plasma exchange/plasmapheresis or immunoglobulin therapies are considered second-line therapies. In a review by Chan, oral prednisolone therapy was frequently reported, the response to corticosteroids was relatively good, and the visual prognosis was better than those of CAR or MAR [1], demonstrating that 8/11 patients with PON exhibited improvement of their visual field to normal or nearly normal after treatments. Therefore, the prognosis of visual function is also thought to be dependent on responsiveness to the treatment of the underlying malignant tumors [4, 5]. There has been a report that plasmapheresis therapy in addition to immunosuppression therapies were effective to preserve visual acuity [11]. In our patient, the first treatment with prednisolone did not influence visual acuity outcomes, and three courses of methylprednisolone pulse treatment were ineffective. However, after switching from gefitinib to osimertinib for systemic treatment with anticancer drugs, the primary lung cancer lesions and systemic metastasis were reduced, and visual acuity was notably improved.

When EGFR-TKI is administered for first-line treatment of EGFR mutation-positive non-small-cell lung cancer, many patients acquire resistance, and it has been reported that the EGFR T790M mutation accounts for approximately 60% of the resistance mechanism [12, 13]. Osimertinib, an EGFR-TKI that is effective despite resistance associated with the T790M mutation, is selected as the second-line treatment. In our patient, bronchoscopy performed during steroid pulse therapy revealed acquisition of the EGFR T790M mutation.

In some cases, loss of visual acuity and visual-field defects can be improved transiently in response to corticosteroid monotherapy; however, it has been suggested that ultimate visual function cannot be preserved unless the tumor is reduced in size early after the onset of optic neuropathy. PON associated with lung adenocarcinoma may have a different mechanism from that of small-cell lung carcinoma, with which anti-CRMP-5 antibodies are associated. However, there are few reports of PON associated with lung adenocarcinoma [10, 14], and a number of autoantibodies related to autoimmune optic neuropathies are yet to be identified. In our patient, the reduced tumor size by osimertinib treatment might lead to a reduction of unknown autoantibody production. Further studies on autoantibodies associated with PON are needed.

## 4. Conclusion

Generally, although loss of vision is improved by immunosuppressive treatment, such as corticosteroids, at an early stage in some cases of PON, improved visual function cannot be preserved without treatment of the underlying tumor. In our patient, changing treatment to osimertinib for primary lung adenocarcinoma resulted in visual acuity improvement. This indicates the importance of thoroughly treating the primary cancer for the treatment of PON.

## Data Availability

All data studied in this case report are included in this report.
